# Effects of emission trading scheme (ETS) on change rate of carbon emission

**DOI:** 10.1038/s41598-023-28154-6

**Published:** 2023-01-17

**Authors:** Hail Jung, Chang-Keun Song

**Affiliations:** 1 Graduate School of Carbon Neutrality, Ulsan National Institute of Science and Technology, Ulsan, Republic of Korea; 2 Department of Urban and Environmental Engineering, Ulsan National Institute of Science and Technology, Ulsan, Republic of Korea

**Keywords:** Climate-change policy, Sustainability

## Abstract

This paper investigates the effects of Emission Trading Scheme (ETS) adoption on the country-level reduction rate of carbon emission. We first used Environmental Kuznets Curve (EKC) tests to group countries into three categories: inverse U-shaped and gamma-shaped EKC for decoupled countries, and a positive linear EKC for non-decoupled countries. We then examined the effectiveness of ETS adoption. We found ETS was effective for both post-industrial and pre-industrial economies. Compared to countries that have not adopted ETS, the carbon emission reduction (increment) rate of decoupled (non-decoupled) countries that have adopted ETS is faster (slower). Furthermore, ETS adoption significantly reduces overall carbon emissions per capita compared to other global events, such as oil crises. The results imply that a market-based mechanism is an effective strategy for achieving sustainable development, thus, providing insights for policymakers and governments to design effective carbon neutrality policies to achieve sustainable development.

## Introduction

Sustainable development is an important goal for human civilization^1^. The United Nations (UN) announced the Sustainable Development Goals (SDGs) framework in 2012 to achieve global sustainable development^2^. Environmental quality plays a pivotal role among the 17 SDGs. Hence, researchers have long investigated methods to improve air and environmental quality.

Although it is necessary to reduce the overall volume of air pollutants, it is also important to ensure economic development. The relationship between environmental quality and a nation’s economic growth has been extensively discussed^3^ based the theoretical foundations originally discussed by Kuznets in 1955. Kuznets^4^ theoretically demonstrated that economic development, often measured by the gross domestic product (GDP) per capita, is positively associated with an increase in environmental degradation (measured as emissions per capita), until a certain developmental threshold is achieved. This hypothesis is the basis of the environmental Kuznets curve (EKC).

When economic development surpasses this threshold, the relationship between development and environmental degradation becomes negative^5^. As such, studies commonly argue that the threshold represents the level of economic prosperity, and when the threshold is exceeded, the nation has the potential to reduce pollution, while enhancing economic welfare^6–8^. Studies show that an inverted U-shaped association between economic development and emission levels is ideal^9,10^, and several studies have empirically examined whether air pollutants, such as carbon dioxide (CO_2_), sulfur dioxide (SO_2_), water pollutants, and other environmental degradation indicators, have an inverted U-shaped relationship^3,11–27^.

Among various air pollutants, reducing carbon emissions is considered important as they are directly linked to several socioeconomic sectors and economic development of a nation^28,29^. Moreover, carbon emissions are closely related to human conditions, such as mortality, deprivation, and leisure^30,31^. Owing to its complexity, increasing carbon emission is a growing concern for policymakers and legislation in most countries, yet studies show that many countries have not passed the tipping point^32–37^. One argument is that EKC depends on national-level factors, such as natural resource availability, technological progress, population, and trade amount^38–40^. Additionally, scholars have long investigated the behavior and factors of carbon emissions in the EKC hypothesis^13,39,41–50^. While prior studies have well examined how trade level between countries, economic status, geopolitical situations affect the EKC, not many has studied how the national-level policies that directly affect the country-level carbon emission have effects on EKC. Thus, countries would need a solid national-level policy to reduce carbon emission as it is related to various industries and sectors.

Thus, policymakers aim to achieve sustainable development by achieving economic growth and reducing carbon emissions simultaneously. Many countries have implemented various policies to effectively control carbon emission. If such policies are effective, then the countries that have implemented them would experience a decrease in carbon emissions compared with countries that have not implemented such policies yet. However, it is difficult to compare the policy effects empirically and statistically between countries. This is even more difficult when the policy content varies across countries. Therefore, we had to choose a regulation that was implemented by multiple countries as a reference policy to divide the country-level sample observations. We thus analyzed the ETS implemented in multiple countries and investigated its effect on the relationship between the GDP per capita and carbon emissions per capita of every country.

ETS is a policy tool used to address greenhouse gas emissions by setting limits on the amount of greenhouse gases that can be emitted by certain industries or sectors. The goal of an ETS is to create an economic incentive for businesses to reduce their greenhouse gas emissions and to shift towards cleaner technologies.

One advantage of an ETS is that it can be a cost-effective way to reduce greenhouse gas emissions by allowing businesses to find the most cost-effective ways to reduce their emissions. This can help to reduce the burden on businesses, as they have flexibility in how they choose to reduce their emissions. Another advantage of an ETS is that it can create a market for greenhouse gas reductions, which can drive innovation and the development of cleaner technologies. This can help to reduce the long-term costs of addressing climate change.

Due to various advantages, many developing and developed countries have enacted national-level ETS. Therefore, using ETS as a policy shock, we divided the samples before and after the turning point and observed the ETS effects. We hypothesize that ETS implementation would have positive effects for both decoupled and non-decoupled countries. The underlying conjecture of our analysis was that, for countries that implemented the ETS before they crossed the turning point, the ETS would reduce the positive coefficient between the GDP per capita and the carbon emissions per capita. However, for countries that implemented the ETS after crossing the turning point, a more negative relationship would occur between the GDP per capita and carbon emissions per capita^43,48,49,51–53^.

In this manner, we have compared data from various studies and drawn valuable inferences regarding the effectiveness of emission reduction policies to mitigate climate change. To test its effects, we first tested EKC model to sample out the valid country seamples, i.e., samples that follow inverted-U shaped relationship between the national economic development and the carbon emission. We first found that 26 countries confirmed the EKC hypothesis. Among them, 16 countries already crossed the turning point (decoupled), and 10 countries have not yet crossed the threshold (non-decoupled). We then employed regression analysis to test the ETS effects. We found that adopting ETS was an effective strategy in reducing the carbon emission rate for both decoupled and non-decoupled countreis. Overall, the aim of our study is to assess the effectiveness of national-level climate change policies on the relationship between economic growth and carbon emissions, thus providing valuable insights that can help countries achieve sustainable development.

Contribution of this paper is clear. We underscore the importance of carbon emission management; carbon emissions are often closely related to human activities and are the primary drivers of climate change^54–58^. However, unlike other air pollutants, carbon emissions in most countries have not yet reached the tipping point of an inverse U-shaped relationship between economic growth and carbon emissions^59^. In this respect, this paper contributes to the EKC literature by providing another factor that may potentially affect the relationship between the economic development and environmental degradation. We show empirical evidence that the adoption of national-level ETS has positive effects for carbon emission change rate for both decoupled and non-decoupled nations. Results imply that the ETS–a market-based mechanism designed to effectively reduce carbon emission while remaining the economic development–is an effective strategy.

Second contribution is on empirically grouping countries according to their decoupling status, to observe the relevance and effects of the level of economic development of individual countries. Our study revealed that ETSs are effective for both developing and developed countries, indicating the effectiveness of market-based mechanisms in reducing carbon emissions, while boosting economic development.

Third, our findings can be useful for policymakers to design effective pathways to achieve carbon neutrality by the second half of the twenty-first century. Based on the results that unidirectionally suggest the positive effects of ETSs, policymakers may consider expanding the regulation so that more entities may join the trading scheme. Notably, our study revealed that countries that have not adopted ETSs may also positively consider it as a viable method to reduce carbon emissions.

## Results

### Environmental Kuznets curve (EKC) across the globe

We deduced that 26 countries confirmed the EKC hypothesis in terms of economic and statistical significance (Table 1). Similar to the results of prior studies, we partially confirmed the EKC hypothesis. Specifically, the EKC hypothesis was confirmed for central and eastern European countries^60–63^, while the results were mixed for western and northern European countries^64,65^, as well as Asian countries^11,66–69^.

**Table 1:** List of (a) Decoupled countries and (b) List of non-decoupled countries.

Country	Decoupled year	Turning point ($ in 1960)	Population (thousands, 2019)	Shape	ETS (year)	OECD	EU
A							
Argentina	1991	1190.04	44,781	None	No	No	No
Australia	2004	4494.99	25,203	Gamma	No	Yes	No
Canada	2007	6196.62	37,411	Gamma	No	Yes	No
Costa Rica	2015	1389.3	5048	Gamma	No	No	No
Czech Republic	2003	1406.32	10,689	Inverse U	Yes (2005)	Yes	Yes
France	2004	5068.2	65,130	Inverse U	Yes (2005)	Yes	Yes
Germany	1990	4340.48	83,517	Inverse U	Yes (2005)	Yes	Yes
Greece	2004	3208.69	10,473	Gamma	Yes (2005)	Yes	Yes
Japan	1973	2359.34	126,860	Gamma	No	No	No
New Zealand	2003	2841.34	4783	Inverse U	Yes (2008)	Yes	No
Russian Federation	2010	1274.02	145,872	None	No	No	No
Singapore	1992	3326.51	5804	Inverse U	No	No	No
Slovak Republic	2003	1340.08	5457	Inverse U	Yes (2005)	Yes	No
Sweden	2003	5596.18	10,036	Inverse U	Yes (2005)	Yes	Yes
Switzerland	2007	9117.96	8591	Inverse U	Yes (2013)	Yes	No
United Kingdom	1987	2902.22	67,530	Inverse U	Yes (2005)	Yes	No

Country	Turning point ($ in 1960)	Population (Thousands, 2019)	ETS (year)	OECD	EU		
B							
Belarus	1071.72	9452	No	No	No		
Brazil	2125.86	211,050	No	No	No		
Bulgaria	1068.45	7000	Yes (2005)	No	Yes		
Chile	3644.88	18,952	No	Yes	No		
Finland	9176.78	5532	Yes (2005)	Yes	Yes		
Korea, Rep	8801.85	51,225	Yes (2015)	Yes	No		
Netherlands	8267.45	17,097	Yes (2005)	Yes	Yes		
Norway	24,186.5	5379	Yes (2005)	Yes	No		
Portugal	3242.45	10,226	Yes (2005)	Yes	Yes		
Saudi Arabia	4094.81	34,269	No	No	No		

Among these countries, 16 have crossed the turning point (decoupled countries), whereas 10 have not (non-decoupled countries). Table 1 a and b show the list of decoupled and non-decoupled countries, respectively. Interestingly, while most countries that crossed the turning point portrayed an inverted U-shaped relationship between the income per capita and carbon emissions, a few exhibited a gamma-shaped relationship.

**Figure 1:**
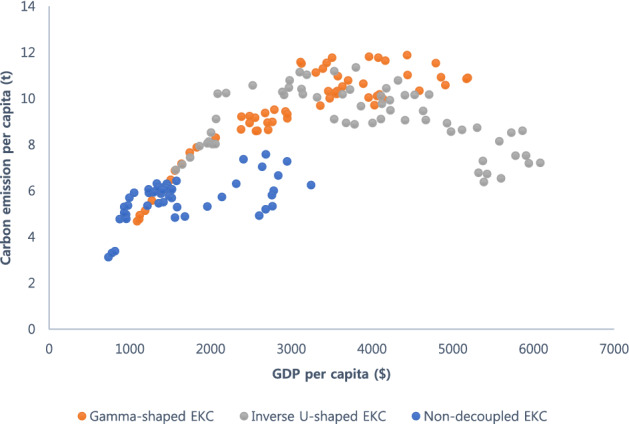
Distribution of the environmental Kuznets curve (EKC) of countries. Gamma-shaped and inverse U-shaped distributions are depicted as orange squares and gray triangles, respectively, and EKC of non-decoupled countries are depicted as blue circles. GDP, gross domestic product.

Consistent with the EKC hypothesis, non-decoupled countries showed a positive relationship between the GDP per capita and the carbon emissions per capita (Fig. 1). Meanwhile, gamma-shaped and inverse U-shaped distributions were observed for decoupled countries. Supplementary Material 1 shows the data of a few countries that have not yet crossed the turning point. Brazil, Saudi Arabia, and the Republic of Korea are examples of countries that are non-decoupled, indicating that their economic growth and carbon emissions have a positive relationship.

The inverse U-shaped EKC indicates an ideal balance between economic development and emission levels. Notably, an inverse U-shaped relationship is ideal for climate-change-sensitive governments to achieve carbon neutrality as these governments aim to reduce carbon emissions, while maintaining positive economic growth. Supplementary Material 1 shows data of countries (e.g., Germany, France, New Zealand, Singapore, Sweden, and Switzerland) that portrayed an inverse-U-shaped relationship.

Simultaneously, we observed a gamma-shaped distribution relationship in the decoupled countries, i.e., these countries failed to reduce carbon emissions, even after they crossed the tipping point and their overall economy improved. While the absolute turning point value varied for each country, we observed a similar trend in most decoupled countries. Countries that seek carbon emission reduction prefer an inverse U-shaped relationship. The gamma-shaped relationship implies that economic development after a threshold is unrelated to the reduction in emissions levels. Supplementary Material 1 presents examples of countries that followed a gamma-shaped distribution, e.g., Australia, Canada, and Japan.

**Figure 2:**
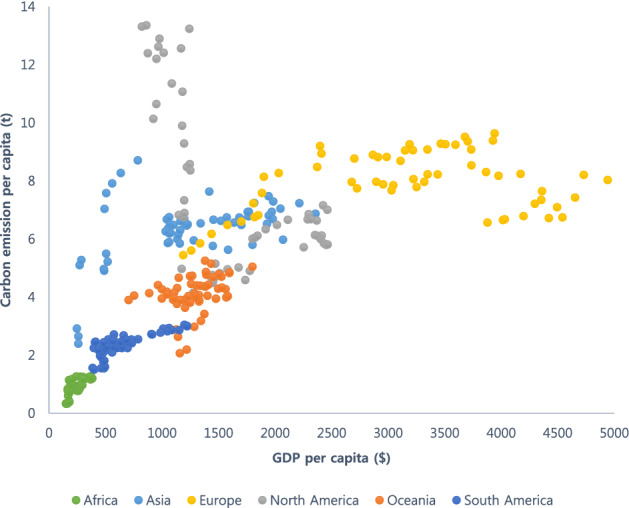
Environmental Kuznets curve **(**EKC) grouped by continents. GDP, gross domestic product.

We further observed these patterns at the continental and regional levels by observing and determining whether the gamma- and inverse U-shaped relationships portrayed continent- or regional-level patterns. The EKC grouped by continent (including only important events) may affect carbon emissions (Fig. 2). Europe was the only continent in which an inverse U-shaped relationship between economic growth and carbon emissions was observed. Moreover, our results revealed that carbon emissions decrease after the GDP per capita crossed approximately USD 3500 (USD value in 1960). Supplementary Material 2a shows a graphical representation of the EKC changes in Europe with respect to different periods (1960–1979, 1980–1999, and 2000–2019). Notably, while economic growth and carbon emissions followed a positive relationship in the 1960s, this trend flattened in the 1980s and subsequently declined sharply in the 2000s. Since then, important events have affected carbon emissions, such as the adoption of the European Union (EU) ETS Phase 1 (2005), Phase 2 (2008), and Phase 3 (2013) and the Paris Agreement (2015).

Additionally, we conducted a univariate analysis to examine the effectiveness of the ETS and compared its effectiveness with other global events. We chose the first (1973) and second (1978) oil shocks as global events that may have significantly affected carbon emissions. This is because oil price fluctuated heavily during the crises, which in turn affected firm-level production and household energy usage. First, we measured and compared differences in the 3-year average emissions before and after the event year for each continent. Panel A and Panel B of Table 2 show the results for the first and second oil shocks, respectively. Carbon emissions before and after the event portrayed no significant difference except for Oceania (Panel A) and North America (Panel B). Panel C shows the results for the three phases of the EU ETS. Similar to the previous two tests, we calculated the 3-year average before and after the enactment of each ETS phase and checked the differences. Regardless of the ETS phase, we observed a significant decrease in carbon emissions. For instance, the carbon emissions per capita reduced by 3.1%, 9.4%, and 10.7% during EU ETS Phases 1, 2, 3, respectively. In terms of the absolute scale, the 3-year average carbon emissions before Phase 1 were 8.894 tons per capita. This reduced to 7.330 tons per capita in the 3-year average carbon emissions after the ETS Phase 3 was implemented.

**Table 2:** Univariate comparison analyzing the effects of oil crises, financial crisis, and ETS adoption.

	3-year average before	3-year average after	Log difference	t-value
Panel A. First oil shock (1973)				
Africa	0.767	0.797	0.038	− 0.205
Asia	7.867	7.386	− 0.063	0.785
Europe	8.297	8.851	0.065	− 0.361
North America	12.126	11.197	− 0.080	0.535
Oceania	4.411	4.863	0.098	− 1.544*
South America	2.179	2.256	0.035	− 0.780
Panel B. Second oil shock (1978)				
Africa	0.828	0.960	0.148	− 0.340
Asia	7.190	6.691	− 0.072	0.983
Europe	8.936	9.359	0.046	− 0.902
North America	10.807	7.693	− 0.340	1.299*
Oceania	4.779	4.457	− 0.070	0.579
South America	2.255	2.465	0.089	− 1.779*
Panel C. EU ETS effects (Phase 1–2005, Phase 2–2008, Phase 3–2013)				
ETS Phase 1	8.894	8.618	− 0.031	1.980**
ETS Phase 2	8.206	7.471	− 0.094	5.270***
ETS Phase 3	8.155	7.330	− 0.107	4.592***

Africa, Asia, Oceania, and South America were yet to reach the turning point and followed a positive relationship between economic growth and carbon emissions. An interesting picture emerged in North America as the carbon emissions decreased sharply at the GDP per capita of USD 1000 and USD 2000 (USD value in 1960) and remained stable thereafter. We analyzed the data for North America further for decades (Supplementary Material 2b) and found that carbon emissions declined sharply in 1960–1979, and subsequently increased slightly, yet consistently. In the 1970s, two oil crises directly affected the U.S.A. causing the manufacturing production to decline sharply, and the stock market to crash^8,70^.

### ETS effects

In this section, we explored the effects of ETS on the EKC for the decoupled, as well as non-decoupled, countries. Specifically, we considered the ETS as a benchmark policy shock for two reasons. First, the ETSs are aimed at regulating firm-level carbon emissions, which are often closely related to human activities, and these emissions are the primary drivers of climate change^7,31,66,71^. Therefore, studying regulations related to carbon emissions may be more helpful than studying those related to other air pollutants. Second, the ETS follows a market-based mechanism in which policymakers hope to effectively reduce emissions without compromising economic growth. In market-based environmental regulation policies, environmental protection may become a business activity, wherein the cost of emissions can be internalized and marketized^72^. Taking full advantage of the ETS, we designed both univariate and multivariate settings to explore the ETS effects on the relationship between carbon emissions and economic growth.

**Figure 3:**
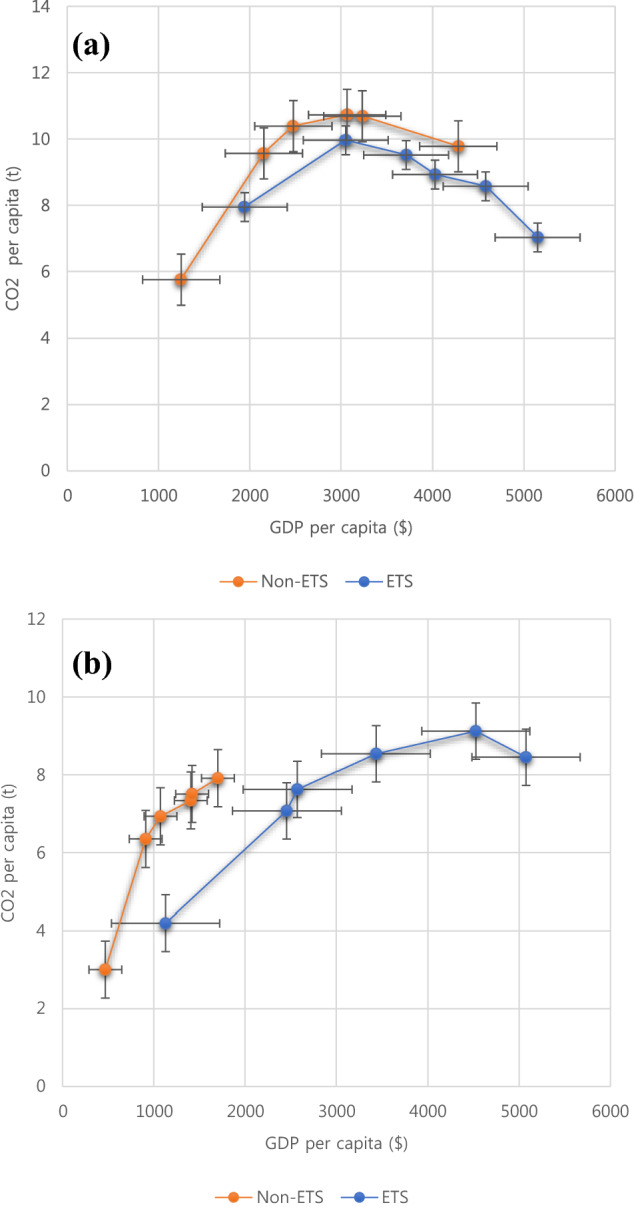
Environmental Kuznets curve (EKC) of decoupled and non-decopled countries classified by emission trading scheme (ETS) participation status and grouped at decade level. (**a**) Decoupled countries; (**b**) non-decoupled countries. GDP, gross domestic product.

Decoupled countries started to adopt the ETS in the mid-2000s. After ETS adoption, carbon emissions per capita started decreasing significantly compared with the countries that did not adopt the ETS (Fig. 3a). Moreover, the decoupled countries that adopted the ETS showed lower carbon emissions. This implies they may have already invested in low-carbon technologies and production methods and employed the ETS to further accelerate emission reduction.

Theoretically, before decoupling occurs, the relationship between the GDP per capita and carbon emissions per capita should be positive^73^. This relationship was observed for non-decoupled countries (Fig. 3b) that did not adopt the ETS. However, the countries that adopted the ETS showed decreasing carbon emissions in the 2010s. Considering that countries began to adopt ETS in the mid-2000s, our results imply that ETS adoption has somehow affected the overall economy of the nation and decreased the emission per capita.

**Figure 4:**
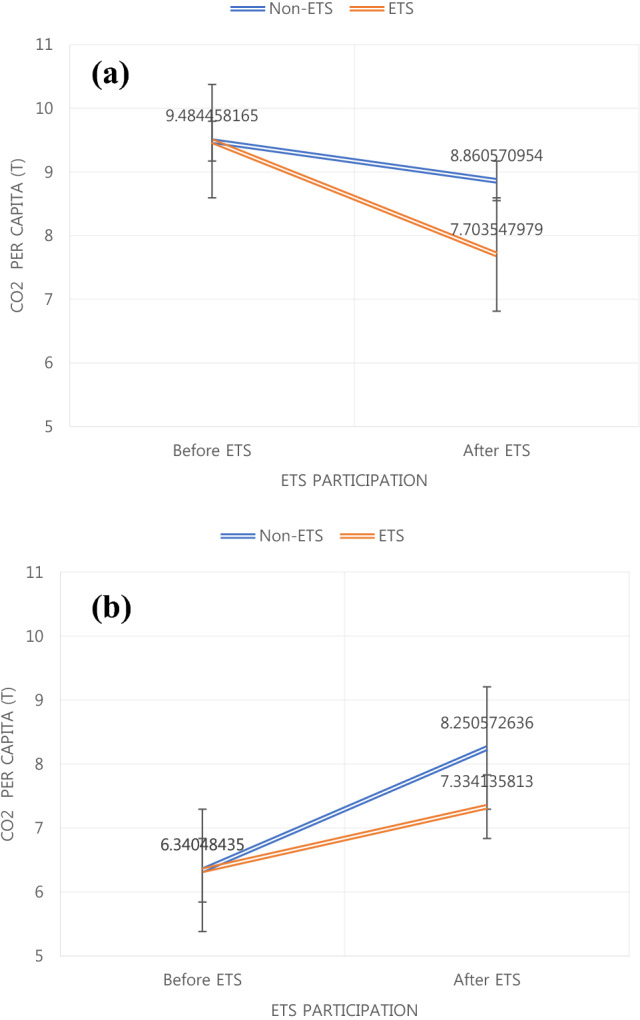
Difference in carbon emission per capita between the countries that have and have not adopted the emission trading scheme (ETS) after and before crossing the turning point. (**a**) Carbon emission per capita in countries after crossing the turning point. (**b**) Carbon emission per capita in countries before crossing the turning point.

Figure 4 presents a more direct comparison of the carbon emission changes after ETS adoption. We averaged the carbon emission per capita of sample countries before and after the ETS adoption. The overall carbon emission per capita was reduced in decoupled samples, which is consistent with the findigns of a prior study^73^. Additionally, the reduction rate was higher for countries that adopted the ETS, implying that effective ETS strategies can help achieve sustainable development. Moreover, consistent with EKC theories, the overall carbon emissions per capita portrayed a significant increase in the countries that did not adopt the ETS. Notably, the carbon emissions of the countries that adopted the ETS increased at a lower rate. Overall, the results implied the significance of ETS in developed and developing countries. However, as developing countries will inevitably emit more carbon while achieving higher productivity, ETS may have slowed the increment rate of carbon emissions in the non-decoupled countries that did not adopt the ETS.

Based on the univariate results, we conducted multivariate regression models to test the ETS effects. Specifically, we used the sample countries for which the EKC condition was applicable. Further, we divided the samples into pre-industrial and post-industrial economies and tested the ETS effects. The empirical results confirmed that ETS adoption, before and after the turning points, had a positive effect on carbon neutrality. The results were consistent, regardless of the model used.

Table 3 shows the results of the regression analysis. Our results revealed that ETS adoption after the turning point further increased the negative relationship between the GDP per capita and the carbon emissions per capita (Column 1 and 2). Moreover, ETS adoption reduced the positive association between economic development and carbon emissions before the turning point (Column 3 and 4). Overall, the estimation results imply that ETS adoption in an early stage of economic development is an effective strategy to reduce the emissions per capita, while ETS adoption after the turning point is also effective in reducing carbon emissions.

**Table 3:** Regression results examining the ETS effects on the relationship between economic development and carbon emissions. Columns (1) and (3) provide the estimation results for ETS participating countries that are decoupled and non-decoupled, respectively. Columns (2) and (4) show the results for non-ETS participants that are decoupled and non-decoupled, respectively.

	Decoupled samples		Non-decoupled samples	
	(1)	(2)	(3)	(4)
Variables	Carbon emission	Carbon emission	Carbon emission	Carbon emission
ln(GDP per capita)	− 0.280***	− 0.101**	0.646***	1.052***
	(− 6.530)	(− 2.396)	(21.010)	(20.232)
ln(population)	0.808**	0.440***	1.359***	− 0.365
	(18.283)	(3.992)	(8.531)	(− 1.043)
ln(trade)	1.192*	0.311	0.599***	0.058
	(9.471)	(1.490)	(8.804)	(0.213)
CPI	0.001	0.002	− 0.001	− 0.001
	(1.194)	(1.692)	(− 0.111)	(− 0.109)
R&D expenses	0.163	0.101**	− 0.17	− 0.07
	(0.932)	(2.177)	(− 0.176)	(− 0.189)
Renewable energy consumption	0.002	0.001***	− 0.11*	− 0.02
	(0.474)	(4.163)	(− 2.112)	(− 0.847)
Consumption expenses	0.002	0.002	0.001	0.006
	(1.106)	(0.978)	(0.309)	(0.402)
Constant	4.442	− 3.081	1.191	− 5.871
Observations	185	120	288	192
Adjusted R-squared	0.179	0.476	0.921	0.652
ETS participation	Yes	No	Yes	No
Year FE	Yes	Yes	Yes	Yes
Country FE	Yes	Yes	Yes	Yes
Two-way FE	Yes	Yes	Yes	Yes

Regression results also imply that ETS acts as a catalyst in altering the relationship between the economic growth and carbon emission. It is true that ETS is not a random experiment, and countries that aredetermined to participate in ETS are usually countries with emission reduction responsibilities. In addition to ETS, these countries will also carry out various other carbon emission reduction policies, including renewable energy subsidies. Therefore, it may be misleading to jump to the conclusion that the statistically significant coefficient solely represents the ETS effects. However, it is also true that countries that are enacting various climate change mitigation strategies including country-level ETS have faster carbon emission reduction, for developed economies and slower carbon emission increment for non-developed economies. With this respect, the coefficients in the analysis may imply that counties’ willingness to reduce carbon emission is somewhat captured through this empirical analysis.

## Conclusion

The Paris Agreement highlights the significance of limiting the average global temperature increase to 1.5 °C above the pre-industrial levels, while pursuing efforts to limit the temperature increase to 1.5 °C^31^. Thus, both developed and developing countries have introduced national-level plans to reduce the carbon emission, possibly down to neutral level. For instance, ICAP reports that more than 70 countries around the globe have pledged to achieve carbon neutrality by 2050^74^, and 138 countries have committed plans for carbon neutrality.

Achieving carbon neutrality by 2050 or 2060 is crucial. However, carbon neutrality cannot be achieved without the participation of governments. Among various policies, previous studies have revealed that ETS or other forms of carbon trading policy are among the most effective in promoting low-carbon and sustainable economic development simultaneously^43,48,49,51–53^. The scheme is considered an effective market-based emission reduction method because of its salient merits, including cost-effectiveness, wide coverage, flexibility, and predictable and transparent market environment^43,54,75^.

In this study, we tested the effects of ETS at the country-level. Specifically, we examined whether ETS had any effect on the relationship between economic growth and climate change. The results confirmed that the policy had a positive effect on both the decoupled and non-decoupled samples. Our findings indicate that, for non-decoupled countries, ETS adoption successfully reduces the magnitude of the positive relationship between the GDP per capita and the carbon emissions per capita. Along with prior studies, we present positive effects of ETS deployment, which may contribute viable guidelines for policymaking.

It is important to understand that no policy framework is absolute or all-encompassing, and ETSs are not an exception. Notably, our study investigated the impact of ETSs on the relationship between economic development and carbon emissions, and we believe that the ETS framework can cater to both developed or developing nations as a baseline approach. Moreover, our study proved that the ETS can be adopted by both developed and developing nations, and most importantly, its effects were evident in both groups. Our study provided an empirical analysis of market-based mechanisms, such as the ETS, and confirms that this scheme may be conducive to achieving carbon neutrality. Furthermore, this study may help government officials and policymakers develop more feasible solutions to reduce carbon emissions, while simultaneously pursuing economic development.

The mechanism connecting the ETS and carbon emission reduction can also be discussed following prior studies. Previous literature has pointed out various mechanisms that drive the carbon emission reduction such as innovation, industrial structure, or industrial chain^76–78^. Our findings also support the previous studies. The mechanism would be that ETS would increase the firm’s burden of carbon emission and may induce firms to innovate their business model from heavy emitting to less emitting, for example, from manufacturing to service industries. Such efforts would in the end change the industrial structure or industrial chain in larger scale^76–78^ and affect the carbon emission amount.

Meanwhile, it is also important to realize that the policy framework should consider the sectoral, societal, and cultural aspects of each nation. We believe that the suggested model could have been more insightful if it could include further national-level characteristics. Inclusion of R&D expenses, consumer expenses, CPI and renewable energy consumption were at best what we could do to understand the dynamics around ETS effects on carbon emission reduction. In this manner, the research could have been more insightful by incorporating unobserved heterogeneities among nations. We leave this question to future research.

## Methods

From the available literature, we used the GDP per capita, GDP per capita squared, log of population, and ratio of trade (imports plus exports scaled by GDP), as independent variables, and carbon emissions, to test the EKC hypothesis for all countries. Following the methods of Churchill et al.^39^, we logged all dependent and independent variables. For full regression specification, please refer to the methodology section. Our results revealed that 16 countries were decoupled; in other words, they crossed the threshold point between economic development and carbon emissions, e.g., Germany, France, Japan, and New Zealand. We also observed that ten countries were non-decoupled, that is, they are yet to cross the turning point, e.g., Brazil, Republic of Korea, and Portugal.

Using the EKC data of the different countries, we examined the effects of climate change policies on the relationship between the economic development and emission levels in each country. Policymakers worldwide have long devoted themselves to designing a policy that would effectively reduce emissions, while not reducing the nation’s production level. Du et al.^72^ suggest that market-based environmental regulation policies can be used to transform environmental protection into a business activity, wherein the cost of emissions can be internalized and marketized. Thus, among various regulations, ETS has been the most commonly implemented measure globally. Notably, an ETS is considered as an approach to reduce the GHGs produced by enterprises, by following market-based mechanisms. ETS is based on the cap-and-trade mechanism, where pollution emitting enterprises are allowed to emit carbon emissions up to a certain threshold (“cap”) and may buy or sell allowances with other enterprises (“trade”). If the firm emits less than the threshold determined by the government, then, the firm may benefit from selling the leftover allowances. However, if the firm emits more than the threshold, the firm would have to purchase the allowances to satisfy the regulation. Obviously, the ETS is a popular policy adopted for climate mitigation^50^.

As a precursor to similar market-based environmental regulations, the EU ETS came into force in 2005 and is the world’s largest well-established and mature carbon market^79^. China, Switzerland, the United Kingdom (UK), and the Republic of Korea have deployed national-level ETSs. In 2013, China established a nationwide ETS in the midst of developing eight regional pilot ETSs. Statistical evidence shows that, between 2008 and 2016, the EU ETS achieved 1 billion tons of carbon emission reduction, compared to other countries without ETS^11^. Moreover, some countries, such as the U.S., have employed ETS at the subnational level, such as California and Massachusetts. Moreover, some developed countries, such as Japan, are actively discussing ETS enactment, and developing countries, such as Thailand, Vietnam, Indonesia, and the Philippines, are developing their ETS systems, or considering employing them. The popularity of ETSs is increasing worldwide, regardless of the country’s economic welfare. Moreover, the increased popularity may help scholars discuss the ETS effects as a climate policy to mitigate climate issues^80,81^. In countries having high carbon emission reduction costs, such as Republic of Korea, the GDP loss can be reduced by 1.42% by employing ETSs^82^. Therefore, it is important to focus on both economic development and climate change. The ICAP Status Report 2021 reported that ETSs regulate over 15% of the global carbon emissions.

In this study, we incorporated the ETS status of various countries around the world; our regression analysis confirmed that ETS adoption during an early stage of economic development can be an effective method to reduce the incremental rate of emissions per capita. Additionally, ETS adoption after decoupling can also accelerate the reduction of carbon emissions. The results indicated that national-level ETS enactment has positive effects for both decoupled and non-decoupled nations, implying that such policies may support an inverted U-shaped association between the economic development and emission level in leading countries.

### Data aggregation

In this study, we used various sources to construct a country-level dataset. For the main dependent variable, carbon emissions per capita, we retrieved the country-level carbon emission data dating back to 1960 from the Global Carbon Project, which is frequently used by other studies to calculate country-level historical carbon emission data^83^. To construct the emissions per capita data, we also used annual population data from each country from the UN World Population Prospects. For the main independent variable, GDP per capita, we acquired the data from the World Bank data source. Population and trade were applied as the control variables; the trade data were calculated as imports plus exports scaled by the GDP. The import and export data were gathered from the World Bank Indicators, which is a data source established by the World Bank. Finally, we used ICAP reports to manually pinpoint the ETS-participating countries and the year they joined the trading scheme. We chose such control variables following prior studies examining the EKC^32–37^. Studies have commonly argued that population and trade are, at minimum, important control variables that may affect the relationship between the economic development and carbon emission^32–37^.

The descriptive analysis results for ETS adopting countries and ETS non-adopting countries are reported in Supplementary Material 3a and 3b. It should be noted that on average, emission per capita and total emission are both larger in ETS non-adopting countries compared to those of ETS adopting countries. However, there are other potential drivers that may affect the relationship between ETS adoption and carbon emission reduction. Thus, we employed multivariate regression models to understand the ETS effects carefully.

### Environmental Kuznets curve (EKC) model construction

To test the EKC hypothesis, first, we empirically examined the EKC model at the country-level. Notably, previous studies used different measures and proxies in the model specification. For example, Churchill et al.^39^ considered the nation’s population and trade activities as the control variables in the EKC regression model, and Zafar et al.^40^ included trade liberalization in the model. Some studies further concluded that the EKC was valid in developed countries, such as those that are a part of the Organization for Economic Co-operation and Development (OECD)^39,84,85^. Therefore, the results were sensitive to the control variables included in the model^71,86^. In accordance with the existing literature, the EKC model was empirically defined, as follows:

$$E=F(Y,{Y}^{2},Z)$$

Where $E$ is the carbon emissions per capita, $Y$ is the GDP per capita, and $Z$ is the set of control variables. Following prior studies, we logged both the dependent and independent variables. The aforementioned formula was thus, expressed as follows:

$${\text{ln}}\left( {CO_{2} } \right)_{i,t} = \beta_{0} + \beta_{1} {\text{ln}}\left( {GDP} \right)_{i,t} + \beta_{2} {\text{ln}}\left( {GDP^{2} } \right)_{i,t} + \beta_{3} {\text{ln}}\left( {TRADE} \right)_{i,t} + \beta_{4} {\text{ln}}\left( {POP} \right)_{i,t} + u_{i,t} ,$$

where ${\mathrm{ln}({CO}_{2})}_{i,t}$ represents the carbon emission per capita of country $i$ in year $t$, ${\mathrm{ln}(GDP)}_{i,t}$ represents the GDP per capita of country $i$ in year $t$, ${\mathrm{ln}({GDP}^{2})}_{i,t}$ and ${\mathrm{ln}(TRADE)}_{i,t}$ represent the trade of country $i$ in year $t$, and ${\mathrm{ln}(POP)}_{i,t}$ represents the population of country $i$ in year $t$.

Following the literature, we performed the regression analysis and tested the significance of the coefficients^73^. For cases where ${\beta }_{1}>0$ and ${\beta }_{2}<0$ and the p-values were below 10%, the EKC hypothesis for the inverse U-shaped relationship between economic growth and carbon emissions was satisfied. When this condition was satisfied, we could calculate the turning point of the GDP (GDP*), as follows:

$${GDP}^{*}={e}^{-\frac{{\beta }_{1}}{2{\beta }_{2}}}$$

Based on the data spanning for 1960–2020, we tested the EKC hypothesis for all the countries, except those that had a GDP per capita below USD 1,000 (U.S. dollar value in 1960) and population below 5 million to reduce bias. Overall, after the procedure, we observed that 26 countries satisfied the EKC hypothesis and portrayed an inverse U-shaped relationship between their economic growth and carbon emissions. Table 1 presents a list of these countries, along with their statistics.

### Regression model construction

We tested the effects of the ETS on the EKC relationship. The samples were divided into decoupled and non-decoupled countries to observe the differences among the samples, and each group was subcategorized according to its ETS participation status. This allowed us to distinctly observe the ETS effects on the decoupled/non-decoupled samples. For the regression model, we further considered possible endogeneity problem thar arises from missing variables. That is, there are other factors such as industrial structure, technical level, consumption structure and others that may potentially affect carbon dioxide emission. Thus further to the trade and population variables controlled in the EKC model, we have collected and gathered following control variables for robustness purposes: R&D expenditure (scaled by GDP), renewable energy consumption (scaled by total energy consumption), consumer price index, consumption expenditure (scaled by GDP). All variables are extracted from the World Bank database.

We have run the regression model with the abovementioned control variables included. The new results are reported in Table 3. The regression model specification was as follows:

$$\begin{aligned} Decoupled \; samples{:} \; \ln \left( {CO_{2} } \right)_{i,t} = & \beta_{0} + \beta_{1} \ln \left( {GDP} \right)_{i,t} + \beta_{2} \ln \left( {TRADE} \right)_{i,t} + \beta_{3} \ln \left( {POP} \right)_{i,t} \\ & + \beta_{4} {\text{CPI}}_{i,t} + \beta_{5} {\text{R}}\& {\text{D}}_{i,t} + \beta_{6} {\text{RENEW}}_{i,t} + \beta_{7} {\text{CONS}}_{i,t} + u_{i,t} , \\ \end{aligned}$$

$$\begin{aligned} Non{\text{-}}decoupled\; samples{:} \; \ln \left( {CO_{2} } \right)_{i,t} = & \beta_{0} + \beta_{1} \ln \left( {GDP} \right)_{i,t} + \beta_{2} \ln \left( {TRADE} \right)_{i,t} + \beta_{3} \ln \left( {POP} \right)_{i,t} + \beta_{4} {\text{CPI}}_{i,t} \\ & + \beta_{5} {\text{R}}\& {\text{D}}_{i,t} + \beta_{6} {\text{RENEW}}_{i,t} + \beta_{7} {\text{CONS}}_{i,t} + u_{i,t} , \\ \end{aligned}$$

where ${\mathrm{ln}({CO}_{2})}_{i,t}$ represents the carbon emission per capita of country $i$ in year $t$, ${\mathrm{ln}(GDP)}_{i,t}$ represents the GDP per capita of country $i$ in year $t$, ${\mathrm{ln}({GDP}^{2})}_{i,t}$ and ${\mathrm{ln}(TRADE)}_{i,t}$ represent the trade of country $i$ in year $t$, and ${\mathrm{ln}(POP)}_{i,t}$ represents the population of country $i$ in year $t$ , ${\mathrm{CPI}}_{i,t}$ represents the Consumer Price Index (CPI) of country $i$ in year $t$ , ${\mathrm{R}\&\mathrm{D}}_{i,t}$ represents R&D expenditure scaled by GDP of country $i$ in year $t$ , ${\mathrm{RENEW}}_{i,t}$ represents the renewable energy consumption scaled by total energy consumption of country $i$ in year $t$ , and ${\mathrm{CONS}}_{i,t}$ represents consumption expenses scaled by GDP of country $i$ in year $t$, respectively.

The regression results are presented in Table 3. Consistent with this hypothesis, we could deduce that ETS adoption before the turning point could reduce the positive association between the economic development and carbon emissions. We also observed that ETS adoption after the turning point further intensifies the negative relationship between the GDP per capita and carbon emissions per capita of a country.

## Supplementary Information

Supplementary Information.

## Data Availability

The dataset analyzed and generated in this study can be requested from the corresponding author upon reasonable request. The raw country-level carbon emission data were derived from the public domain: our world in data (https://ourworldindata.org/co2-and-other-greenhouse-gas-emissions?source=post_page-----47fa6c394991). The other data are publicly available.

## Code availability

The raw data, SAS code (for merging), and STATA code (for running the regression) can be requested from the corresponding author.
