# Supplementary material for: Effects of emission trading scheme (ETS) on change rate of carbon emission
Source: Sci Rep. 2023 Jan 17;13:912. doi: 10.1038/s41598-023-28154-6 (PMC9845311; doi:10.1038/s41598-023-28154-6)
Supplement: Supplementary file 1 — Supplementary Information. [file 41598_2023_28154_MOESM1_ESM.docx]

**Supplementary Material**

**Supplementary Material 1:** EKC samples of countries following gamma-shaped and inverse U-shaped distributions, and samples of non-decoupled countries. GDP stands for gross domestic product.

| **Decoupled countries** | | **Non-decoupled countries** |
| --- | --- | --- |
| **Gamma-shaped** | **Inverse U-shaped** |  |
| Australia | France | Korea, Rep. |
| Canada | Singapore | Brazil |
| Japan | Sweden | Saudi Arabia |

**Supplementary Material 2a:** Environmental Kuznets curve (EKC) changes in Europe for different periods. GDP stands for gross domestic product.

**Supplementary Material 2b:** Environmental Kuznets curve (EKC) changes in North America with respect to different periods. GDP stands for gross domestic product.

**Supplementary Material 3a:** Descriptive statistics for ETS adopting countries

| Variables | Obs | Mean | Std. Dev. | Min. | Max. |
| --- | --- | --- | --- | --- | --- |
| CO2 per capita | 473 | 8.659 | 3.167 | 2.011 | 17.846 |
| CO2 total | 473 | 2.540e+08 | 3.050e+08 | 11755131 | 1.063e+09 |
| GDP per capita | 473 | 3910.246 | 2240.424 | 612.002 | 10565.159 |
| Population | 473 | 26500000 | 27900000 | 2627000 | 81646000 |
| Trade | 473 | .676 | .338 | .236 | 1.814 |
| CPI | 473 | 75.278 | 45.79 | .783 | 201.239 |
| R&D expense | 473 | .391 | .302 | .038 | 1.246 |
| Renewable energy | 473 | 18.729 | 27.244 | .05 | 88.072 |
| Consumption expense | 473 | 84.232 | 15.054 | 54.6 | 115.503 |

**Supplementary Material 3b:** Descriptive statistics for ETS non-adopting countries

| Variables | Obs | Mean | Std. Dev. | Min. | Max. |
| --- | --- | --- | --- | --- | --- |
| CO2 per capita | 312 | 9.484 | 5.792 | .404 | 18.958 |
| CO2 total | 312 | 5.080e+08 | 6.080e+08 | 672592 | 2.357e+09 |
| GDP per capita | 312 | 2807.953 | 2145.069 | 327.833 | 8153.772 |
| Population | 312 | 48800000 | 52600000 | 1540000 | 1.480e+08 |
| Trade | 312 | .882 | 1.079 | .115 | 4.109 |
| CPI | 312 | 63.27 | 42.446 | .015 | 161.221 |
| R&D expense | 312 | 1.94 | 1.145 | .168 | 3.705 |
| Renewable energy | 312 | 45.327 | 25.68 | .44 | 94.852 |
| Consumption expense | 312 | 83.21 | 20.531 | 47.438 | 190.103 |
